# Prevalence of hereditary transthyretin amyloid polyneuropathy in idiopathic progressive neuropathy in conurban areas

**DOI:** 10.1186/s42466-019-0035-z

**Published:** 2019-09-18

**Authors:** Andreas Thimm, Saskia Bolz, Michael Fleischer, Benjamin Stolte, Sebastian Wurthmann, Andreas Totzeck, Alexander Carpinteiro, Peter Luedike, Maria Papathanasiou, Christoph Rischpler, Ken Herrmann, Tienush Rassaf, Lars Steinmüller-Magin, Christoph Kleinschnitz, Tim Hagenacker

**Affiliations:** 1 Department of Neurology, University Hospital Essen, Essen, Germany; 2 Department of Cardiology and Vascular Medicine, West German Heart and Vascular Center, University Hospital Essen, Essen, Germany; 3 Department of Nuclear Medicine, University Hospital Essen, Essen, Germany; 4 Institute of Laboratory Medicine and Human Genetics, Singen, Germany; 5 Department of Hematology, University Hospital Essen, Essen, Germany; 6 West-German Amyloidosis NETwork, University Hospital Essen, Essen, Germany

**Keywords:** TTR, Amyloidosis, Cardiomyopathy, Epidemiology, Genotype-phenotype correlation

## Abstract

**Background:**

Hereditary transthyretin amyloidosis (ATTR amyloidosis) is a rare, genetically heterogenous, and clinically variable autosomal dominant disease that severely reduces life expectancy. As treatment options grow, a proper diagnostic approach is mandatory especially in non-endemic regions with diverse genetic backgrounds.

**Methods:**

We examined 102 neuropathy patients at a German neuromuscular centre. Common causes of polyneuropathy were ruled out by medical history and extensive laboratory testing to define a cohort of patients with progressive polyneuropathy classified as idiopathic. Molecular genetic testing of the entire *TTR* gene was performed, and the detected amyloidogenic and non-amyloidogenic variants were associated with the observed clinical phenotypes and results of prior diagnostic testing.

**Results:**

Two of 102 patients tested positive for amyloidogenic mutations (p.Ile127Val and p.Glu81Lys), while a variant of unknown significance, p.Glu26Ser, was found in 10 cases. In both positive cases, previous negative biopsy results were proved by gene sequencing to be false negative. In case of the p.Glu81Lys mutation we detected clinical presentation (combination of severe polyneuropathy and cardiomyopathy), ethnic background (patient of polish origin, mutation only reported in Japanese families before), and disease course clearly differed from well-known cases of the same mutation in the literature.

**Conclusions:**

In conclusion, transthyretin hereditary amyloid polyneuropathy (ATTR-PN) should be considered in cases of otherwise idiopathic polyneuropathy. Sequencing of the four exons of the *TTR* gene should be considered the key step in diagnosis, while tissue biopsy possibly leads to false negative results.

## Background

Hereditary transthyretin amyloidosis is a rare, potentially life-threatening autosomal-dominant disease characterised by extracellular deposition of amyloid fibrils composed of transthyretin (TTR). TTR is a homotetrameric plasma protein transporting thyroxine and retinol binding protein-vitamin A complex [1]. It is mainly synthesised in the liver and to a far lesser extent in the choroid plexus and the retinal pigment epithelium [2]. Tetramer dissociation represents the key step in the formation of misfolded oligomers and amyloid fibrils, which exert various toxic effects on surrounding tissues [3–5]. To date, more than 120 amyloidogenic mutations in the *TTR* gene have been identified. Among the main clinical phenotypes arising from those mutations, hereditary amyloid polyneuropathy (ATTR-PN) and amyloid cardiomyopathy [6] are the most common.

In endemic regions, especially particular areas in Portugal and Sweden prevalence rates of up to 1 in 1000 [7, 8] can be found. Here, ATTR-PN often presents as a rapid, progressive, and irreversible length-dependent sensorimotor and autonomic neuropathy [9] with early small fibre dysfunction as one clinical red flag among others, such as bilateral carpal tunnel syndrome [10]. In fact, the pattern of clinical presentation depends on several factors, including genotype, the patient’s geographical origin and age at symptom onset [11–14]. Genetic heterogeneity, clinical variability, and generally low prevalence frequently results in a delay in diagnosis of several years, especially in patients with a negative family history [15, 16]. However, because of the severe natural course of ATTR-PN in endemic regions leading to disability and death within ten years, early diagnosis and treatment initiation are mandatory. This early diagnosis is of great importance in light of recent emerging treatment options apart from liver transplantation, which has been the standard therapeutic strategy since 1990. In 2011, the first transthyretin tetramer stabilising oral agent, Tafamidis, was approved in the European Union [17], while disease-modifying treatment by gene silencing emerged as another promising approach, with patisiran and inotersen being recently approved for ATTR-PN treatment [18, 19].

Here, we performed genetic testing in 102 patients suffering from a polyneuropathy otherwise classified as idiopathic from a university outpatient clinic to determine the frequency of amyloidogenic mutations in the *TTR* gene and their correlation with clinical phenotypes in a clearly characterised patient cohort in a German conurbation.

## Methods

### Patient selection

We collected data from 102 patients between 2015 and 2018 with electrophysiologically and clinically confirmed idiopathic large fibre neuropathy or clinically and bioptically confirmed small fibre neuropathy presenting at our neuromuscular outpatient clinic. To rule out common causes of peripheral neuropathy, routine laboratory testing was performed including HbA1c, vitamin B1, B6, B12, folic acid, TSH, ANA, ANCA, rheumatoid factor, hepatitis B and C serology, cryoglobulins, immunofixation, glomerular filtration rate, and serum electrophoresis. Alcohol abuse and exposure to other toxic agents were excluded by thorough medical history. Only patients without abnormalities in the above-mentioned parameters were included. Considering expected clinical variability of ATTR-PN in a non-endemic area, we defined no exclusion criteria for clinical presentation or electrophysiological type of neuropathy (primary axonal, demyelinating or both) to avoid selection bias. The study was approved by the ethics committee of the University Duisburg-Essen, Germany. All subjects gave their informed consent prior to participation.

### Performed examinations

All patients included in the study were subjected to a detailed medical history including age at symptom onset, clinical course, previous out- and inpatient referrals, previous diagnostic findings and treatments, ethnic origin and family history as well as a complete physical examination. Moreover, nerve conduction studies (NCS) were performed to specify neuropathy subtypes. NCS comprised measurements of motor and sensory nerves of upper and lower extremities on both sides. Distal motor latency, nerve conduction velocity, motor and sensory amplitude, and F-wave latency were assessed. Cerebrospinal fluid was obtained by lumbar puncture and tested for protein level, cell count, and infectious agents in case of pleocytosis. Finally, we collected blood samples from each patient for genetic testing.

### Molecular genetic analysis

After obtaining the patient’s written informed consent, genomic DNA from total peripheral blood samples was used for genetic testing. All four exons of the *TTR* gene were amplified and sequenced by means of a single-gene Sanger sequencing. The detected variants on protein level are named according to Human Genome Variation Society nomenclature guidelines including the signal peptide (e.g. p.Val50Met). As nomenclature traditionally used to describe those mutations did not follow the general rule of starting with methionine as the translation initator, the traditional mutation names (e.g. p.Val30Met) are given in brackets where they appear for the first time.

## Results

**Fig. 1:**
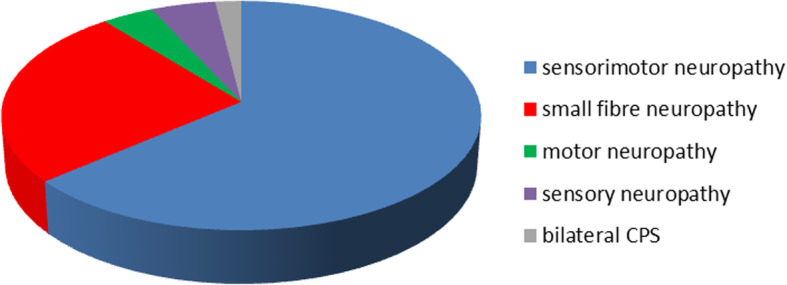
Neuropathy subtypes (in percent)

We collected data from a cohort of 102 patients, 65 with a sensorimotor neuropathy, four with a pure motor neuropathy, five with a pure sensory neuropathy, two with bilateral carpal tunnel syndrome (CTS), and 26 with a small fibre neuropathy confirmed by skin biopsy (s. Figure 1). In the majority of patients, we detected two wild-type alleles with no genetic variants in coding regions of the *TTR* gene apparent. Nevertheless, in ten patients, a heterozygous sequence variant in exon 2, p.Gly26Ser (Gly6Ser), was detected. Four of those patients belonged to the above-mentioned sensorimotor neuropathy subgroup, four had a pure motor neuropathy, and two had an exclusive small fibre involvement. Apart from the p.Gly26Ser polymorphism, there were five patients with single nucleotide variants in non-coding regions of the *TTR* gene (s. Table 1).

**Table 1:** TTR gene variants detected in 102 patients with polyneuropathy

*N* = 102	p.Ile127Val	p.Glu81Lys	p.Gly26Ser	p.Thr139Met	c.337-18G > C
sensorimotor neuropathy	1	1	4	1	4
SFN	–	–	4	–	1
motor neuropathy	–	–	2	–	–
sensory neuropathy	–	–	–	–	–
bilateral CTS	–	–	–	–	–
Sum	1	1	10	1	5

Two of 102 patients tested positive for amyloidogenic mutations in the *TTR* gene, thereby confirming diagnosis of ATTR-PN. In both cases, ATTR-PN was not suspected for years due to symptomatology not explicitly suggestive of amyloidosis or to an incomplete diagnostic approach. Both cases differed in type of mutation, manifestation, course of the disease, and the patient’s ethnic background.

### Case 1

A 71-year-old male originally from Kazakhstan presented with symptoms already progressive for 2 years, especially burning pain, tingling paraesthesia, and disturbed thermosensation in his feet and distal lower legs and also gait and postural disturbance. In the last 18 months before presentation at our outpatient clinic, he had lost 17 kg of body weight without any obvious reason. Approximately 2 years prior, a renal cell carcinoma had been treated by partial nephrectomy. Additionally, magnetic resonance imaging had raised suspicion of hypertrophic cardiomyopathy. In the patient’s family history, there was no evidence of neurologic or severe disabling diseases.

In the neurological examination, sensory deficits in sensations of touch, temperature and vibration and weak tendon reflexes in the distal lower extremities were detected, whereas weakness and muscular atrophy were absent. However, prominent signs of disturbance of extrapyramidal motor function such as rigidity and Parkinsonian gait were observed, prompting us to initiate dopaminergic treatment.NCS showed a primarily axonal pattern of sensorimotor polyneuropathy, while routine laboratory testing including cerebrospinal fluid and onconeural antibody testing had revealed no abnormalities. A biopsy of the sural nerve had shown a severe neuropathy without providing insight into its specific pathogenesis, and amyloid deposits had not been detected in congo red staining (including fluorescence) as well as in an additionally conducted salivary gland biopsy. Dopamine transporter (DAT) SPECT (single photon emission computed tomography) imaging and the patient’s response to dopaminergic treatment supported the diagnosis of comorbid parkinson’s disease.

Molecular genetic analysis showed the well-known amyloidogenic mutation, p.Ile127Val (Ile107Val), in exon 4 of the *TTR* gene, confirming diagnosis of ATTR-PN. Cardiac involvement was verified by myocardial biopsy. Finally, two and a half years after symptom onset, treatment with tafamidis was initiated and continued for almost 2 years now. Molecular genetic testing revealed the patient’s daughter to be an asymptomatic carrier of the same amyloidogenic mutation, whereas his son had no mutation.

### Case 2

A 62-year-old female of Polish origin presented with burning pain and tingling dysesthesias in both hands and feet as well as progressive numbness in the distal upper and lower extremities that she had first experienced 5 years prior. Over the course of disease, she had developed a severe gait disorder, a bladder dysfunction and alternating episodes of constipation and diarrhoea, the latter resulting in considerable weight loss. Her body mass index was reduced to 18 kg/m^2^. The family history was negative for similar symptoms or neurologic disorders. Previously, the patient was treated with intravenous immunoglobulins assuming an underlying inflammatory neuropathy, as NCS results fulfilled the electrophysiological EFNS/PNS (European Federation of Neurological Societies/Peripheral Nerve Society) criteria for chronic inflammatory demyelinating polyradiculoneuropathy (CIDP). The treatment had no impact on disease progression, and even an interfering somatoform disorder was suggested.

Physical examination revealed slight bilateral paresis of plantar flexion (MRC 4/5), a prominent atrophy of both quadricep muscles, most pronounced on the right side, and distal symmetrical hypoesthesia for touch, thermal stimuli, and vibration in the lower extremities, but pain sensation was not impaired. Tendon reflexes in both legs were decreased, and the patient presented with a mild afferent gait ataxia. The upper extremities showed no weakness and only a slight thermal hypoesthesia in both hands and dysesthesias.

Thorough NCS revealed a severe sensorimotor polyneuropathy with features of both axonal damage and demyelination. EMG recordings did not show pathological spontaneous activity but a high degree of polyphasic potentials and a neurogenic recruitment pattern of motor unit potentials. Using the Ewing test, a reduction in heart rate variability indicating autonomic neuropathy was detected. As outlined above, routine laboratory testing and investigation of cerebrospinal fluid yielded no decisive results. A rectal biopsy was negative for amyloid deposits.

**Fig. 2:**
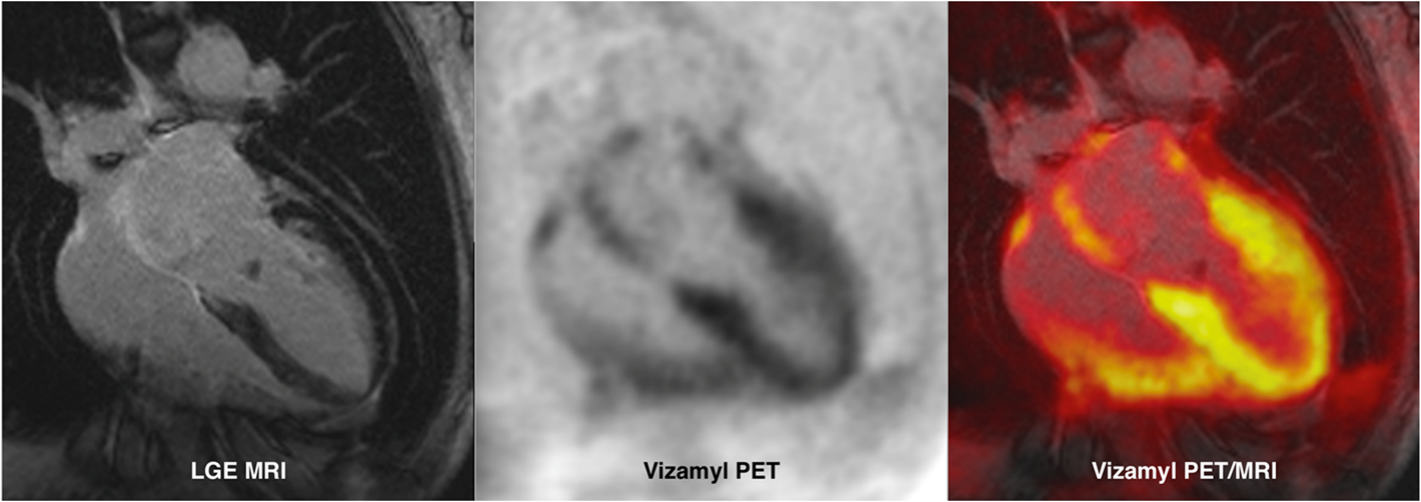
Cardiac amyloid PET/MRI of a patient with hereditary ATTR amyloidosis (case 2): Late gadolinium enhancement (LGE) MRI demonstrates a thickened septal wall, diffuse LGE of the ventricular walls and contrast enhancement of the atria (left panel). On F-18 flutemetamol (Vizamyl) PET scan, moderate to intense tracer accumulation in the right and left ventricular walls as well as in the atria was observed (middle panel). The right panel demonstrates the overlay of amyloid PET and LGE MRI

Molecular genetic analysis revealed a pathogenic mutation in exon 3 of the *TTR* gene, p.Glu81Lys (Glu61Lys), which was previously only reported in Japanese patients [20]. One of the patient’s sons tested positive for the abovementioned amyloidogenic mutation, and the other son refused genetic testing. Finally, with a delay of more than 5 years from symptom onset, treatment with tafamidis was initiated and continued for approximately 4 years. Recently, the treatment was switched to patisiran on both the patient’s wish and the physicians recommendation based on individual indications and contra-indications. Meanwhile a growing cardiomyopathy was detected. (s. Figure 2).

## Discussion

In our study in a German neuromuscular centre within a metropolitan area with patients of diverse genetic backgrounds due to migration, we demonstrated that ATTR-PN is probably not as rare as it is often thought to be in idiopathic polyneuropathy patients in comparable settings. Considering clinical red flags for ATTR-PN such as autonomic dysfunction, weight loss and rather rapidly progressive disease course [10, 21] as selection criteria, positive genetic testing rates can be assumed to be even higher. On the other hand, in case of our first presented patient with genetically confirmed ATTR-PN (case 1) amyloidosis was certainly not evident at first glance as clinical presentation was partially dominated by signs of a Parkinsonian syndrome. Furthermore, sural nerve and salivary gland biopsy were both false negatives, similar to the rectal biopsy in the second described case (case 2) of genetically confirmed ATTR-PN. As a consequence, the importance of molecular genetic analysis to diagnose transthyretin amyloidosis and its superiority over biopsy cannot be overemphasised. ATTR-PN should be considered even in cases of idiopathic polyneuropathy with partially misleading symptomatology due to the wide spectrum of possible clinical presentations [22–24], including treatment-refractory CIDP as the most frequent misdiagnosis [25] but also less frequent phenotypes such as amyotrophic lateral sclerosis-mimicking motor neuropathy [26]. Furthermore, our results show that negative family history cannot exclude ATTR-PN, which is in line with known epidemiologic data revealing a positive family history in less than 50% of all cases of late-onset ATTR-PN in non-endemic regions [27].

Our findings strongly support the notion that, at least in non-endemic areas, proper diagnostic testing requires sequencing of all exons of the *TTR* gene because of genetic heterogeneity as underscored by the amyloidogenic p.Glu81Lys point mutation in our Polish female patient (case 2) that was previously only reported in Japanese patients [20, 28]. Intronic variants in TTR have never been described as disease-causing so far, which appears reasonable considering the pathomechanisms. As regulatory variants cannot influence the protein stability, no toxic gain-of-function/amyloidogenic effect is to be expected. Nonetheless, genotype-phenotype correlations remain challenging. Even among carriers of identical mutations within one family, clinical manifestations may vary, possibly because of posttranslational modifications. The main clinical features in the case of the p.Glu81Lys mutation in the reported Japanese families were either primarily late-onset sensorimotor polyneuropathy [20] or cardiomyopathy and bilateral carpal tunnel syndrome [28], whereas our Polish patient exhibited a mixture of neuropathic and cardiac manifestations. The second detected variant, p.Ile127Val, was previously described in American patients of German descent [29, 30] with a predominantly neuropathic phenotype similar to our findings.

The significance of the p.Gly26Ser variant in terms of amyloid formation remains to be determined. There is some evidence that in cases of bioptically confirmed small fibre neuropathy with autonomic symptoms, p.Gly26Ser frequency might be significantly higher, up to 40%, than in healthy controls [31], suggesting an association between this variant and the development of neuropathy. On the other hand, in a cohort of *TTR* wild-type cardiomyopathy patients, the p.Gly26Ser polymorphism was found in 7% of subjects and 12% of healthy controls [32], prompting the authors to discuss the genetic variant as a protective factor. Regarding the latter results, the p.Gly26Ser frequency of 10% in our study is not likely to be a risk factor for neuropathy development, although to our knowledge there has not been functional testing of amyloidogenicity of this variant.

## Conclusions

In conclusion, in conurban areas in non-endemic regions, molecular genetic analysis for ATTR-PN should be considered in cases of idiopathic polyneuropathy regardless of family history and biopsy results to initiate proper treatment of a potentially life-threatening disease.

## Data Availability

The datasets analysed during the current study are available from the corresponding author on reasonable request.

## Abbreviations

ANA: Antinuclear antibodies
ANCA: Anti-neutrophil cytoplasmic antibodies
ATTR-PN: Transthyretin hereditary amyloid polyneuropathy
CIDP: Chronic inflammatory demyelinating polyradiculoneuropathy
DAT: Dopamine transporter
EMG: Electromyography
HbA1c: Glycated hemoglobin
MRC: Medical Research Council
NCS: Nerve conduction studies
SPECT: Single photon emission computed tomography
TSH: Thyroid-stimulating hormone
TTR: Transthyretin
